# Exploring registered nurses’ experiences and perceptions of nurse manager leadership and its impact on work engagement: A qualitative study set in Saudi Arabia

**DOI:** 10.1371/journal.pone.0340471

**Published:** 2026-02-13

**Authors:** Amal Alluhaybi, Kim Usher, Joanne Durkin, Amanda Wilson

**Affiliations:** 1 Faculty of Nursing, Umm Al Qura University, Makkah, Saudi Arabia; 2 School of Nursing and Midwifery, Faculty of Health, the University of Technology Sydney, Sydney, New South Wales, Australia; 3 School of Health, University of New England, Armidale, New South Wales, Australia; 4 School of Nursing and Midwifery, University of Newcastle, Newcastle, New South Wales, Australia; Mutah University, JORDAN

## Abstract

**Aim:**

To explore nurses’ perceptions of the impact of nurse managers’ leadership practices on work engagement.

**Design:**

A qualitative descriptive study.

**Method:**

Thirteen (13) registered nurses were recruited from different departments in four referral hospitals in Saudi Arabia. Data were collected using semi-structured interviews, and thematic analysis was conducted using NVivo-12 software. The findings were reported following the COREQ checklist.

**Results:**

Key themes included the impact of (1) fair, equitable, and culturally competent nurse managers, (2) effective communication styles by nurse managers, and (3) supportive and collaborative nurse managers. These elements have a positive impact on the level of nurse work engagement.

**Conclusion:**

Nurse managers’ leadership and interaction with their staff can impact nurses’ sense of engagement in their work. Engaged nurses demonstrate higher levels of motivation, job satisfaction, and commitment, which can enhance patient care quality and workplace efficiency. The findings emphasise the importance of key leadership qualities, including cultural competence, effective communication, and supportive leadership, in fostering nurse engagement. These competencies not only improve staff motivation but also contribute to better patient outcomes. Future research should further explore the intersection of leadership practices and diverse cultural contexts to deepen the understanding of how culturally responsive leadership can enhance nurse engagement across various settings.

**Implications:**

To enhance nurse engagement and patient care outcomes, nurse managers should develop their leadership skills, particularly in fairness, cultural competence, and communication. Nursing policymakers should prioritise these competencies in leadership training to foster a supportive workplace. Incorporating cultural competence into leadership training policies will help retain a motivated and diverse nursing workforce. Future research should explore interventions that further develop these leadership skills to improve nurse engagement and patient outcomes.

## 1. Introduction

Registered Nurses constitute the largest healthcare workforce globally and are often the primary caregivers in the healthcare system [1]. Nurses contribute significantly to delivering high-quality healthcare services and improving health outcomes for people, families, and communities [2,3]. Most countries have substantial shortages of nurses, making up 50% of the global healthcare workforce’s shortfalls [4,5]. These shortages are exacerbated by a high number of retiring nurses and the increasingly complex healthcare needs of patients [6–8], which contributes to an elevated nursing workload [9]. As a result, staffing shortages negatively affect nurses’ work-life balance, job performance, and the overall quality of patient care [6,10,11].

Addressing these shortages requires not only attracting new nurses but also retaining the current workforce through fostering higher levels of work engagement [12]. Work engagement, a key factor in both patient care and staff well-being [13], is defined as a positive state of mind characterised by three dimensions: vigour, dedication, and absorption [14,15]. Vigour refers to the mental and physical resilience necessary for nurses to perform their duties effectively, dedication reflects their strong commitment to patient care and the profession, and absorption refers to the full immersion in work tasks [15]. Encouraging high levels of work engagement leads to more motivated and committed nurses, who are less likely to leave their positions, ultimately contributing to workforce stability and improved patient outcomes [16,17].

Managers play a crucial role in influencing the engagement levels of their staff [15,18]. Effective leadership fosters a supportive environment that promotes professional growth and recognises nurses’ contributions., which are vital for organisational success [19,20]. While previous research has examined the relationship between leadership and work engagement, most studies have relied on quantitative surveys that provide broad statistical trends but often fail to capture the complexities of leadership behaviours within specific cultural and organisational settings [21,22]. There is a lack of qualitative research examining how leadership influences nurse engagement in Saudi Arabia, particularly within its culturally diverse workforce. This study addresses this gap by employing qualitative methods to explore how Saudi and non-Saudi nurses perceive leadership and engagement, offering insights beyond numerical data.

Qualitative research offers deeper insights into participants’ personal experiences, thoughts, and attitudes, which standard surveys and questionnaires may not fully capture [21,23]. In-depth interviews, in particular, are a valuable tool for exploring the complexities behind survey findings and understanding these relationships at a more profound level [24]. This study uniquely addresses these gaps by exploring the perspectives of both Saudi and non-Saudi nurses, shedding light on how cultural differences influence perceptions of leadership and work engagement. By employing qualitative methods, the study provides rich insights into these cultural and organisational dynamics, contributing actionable findings tailored to the goals of Saudi Vision 2030.

In Saudi Arabia, the healthcare system is undergoing a significant transformation as part of the Saudi Vision 2030 initiative, which aims to reduce the country’s reliance on oil, diversify the economy, and expand public service sectors such as healthcare, education, infrastructure, and tourism [25]. Similar to global trends, Saudi Arabia faces nursing shortages, which Saudi Vision 2030 seeks to address by making nursing a more attractive and sustainable career for Saudi nationals, thereby reducing the country’s dependence on expatriate workers. Nurse leadership is critical in this context, as effective leadership supports the creation of a resilient, efficient, and patient-centred healthcare system [26]. Enhancing nurse engagement aligns with the objectives of Vision 2030, as engaged nurses are more likely to remain in their positions, contributing to workforce stability and better patient outcomes.

Despite the global and local importance of nurse manager leadership in fostering work engagement, little is known about how leadership practice influences nurse engagement in Saudi Arabia. This study seeks to address a gap in the literature by exploring how nurse manager leadership affects work engagement from the perspectives of nurses in Saudi Arabia. Specifically, the study focuses on two key research questions:

1. How do registered nurses perceive the impact of nurse manager leadership behaviours on their work engagement?
2. What leadership qualities do registered nurses believe are most influential in promoting their engagement?

## 2. Methods

### 2.1 Aim

To explore registered nurses’ perceptions of how nurse manager leadership impacts work engagement.

### 2.2 Design

A descriptive qualitative design was employed. Semi-structured interviews were conducted from May to July of 2023 by AM. The study adheres to the COREQ recommendations for reporting qualitative research [27], ensuring transparency and rigour in the study’s design, data collection, and analysis processes.

### 2.3 Participants

A purposive sampling technique was used to select knowledgeable and experienced interview participants who were suitable for qualitative research interviews. The study participants were registered nurses, both expatriates and locals, working in Saudi Arabia with at least one year experience as a registered nurse and not employed in a managerial role. They were recruited from four referral hospitals in western Saudi Arabia. The recruitment period for this study was from [15/05/2023] to [15/07/2023]. Fifty participants indicated interest in being interviewed in the initial phase, but not all responded to follow-up communication. Thirteen nurses gave informed consent and participated in interviews. Data collection was concluded at this point as no new information was being heard.

### 2.4 Data collection

Data for the qualitative phase were collected through semi-structured interviews on the Zoom platform by AM. The interview questions for the qualitative phase were developed based on insights from the preceding quantitative phase, which identified significant relationships between leadership styles and work engagement. These findings informed the creation of open-ended questions designed to explore participants’ perceptions and experiences with leadership behaviours in greater depth. Research team consultations further refined the questions to ensure they aligned with the study’s objectives.

Interviews were conducted in Arabic with participants whose first language was Arabic (n = 11) and in English with non-Arabic speakers (n = 2). The interviews ranged in length from 25 to 60 minutes, averaging 39 minutes. Research notes, taken during and after the interviews, were later reviewed with the research team to determine the point of data saturation, which was achieved when similar themes emerged, and no new ideas were revealed. Data collection concluded after 13 interviews, guided by the principle of data saturation. Saturation was reached when no new themes, insights, or significant variations emerged, ensuring that the dataset was comprehensive and rich enough to address the research objectives. This approach adheres to qualitative research best practices, balancing the depth and manageability of the data [28,29].

### 2.5 Data analysis

All interviews were digitally recorded and transcribed verbatim by AM. The transcriptions were then checked for accuracy by AM, who reviewed the English transcripts while listening and re-listening to the audio recordings. This process allowed for corrections and the capture of initial thoughts. A bilingual translator translated the Arabic interviews into English, and AM verified the translations to ensure accuracy without altering the meaning.

Data were analysed using the thematic analysis guidelines proposed by Braun and Clarke [30] using NVivo-12 software [31]. The methodological framework comprised six phases: becoming familiarised with the data, generating initial codes, identifying themes, reviewing themes, defining, and finalising themes, and documenting the process. Each phase was systematically applied as follows:

1. Familiarization with the Data: The researchers repeatedly read the transcripts and listened to the audio recordings to gain a deep understanding of the data. Memos and annotations were used to document significant insights and preliminary observations.
2. Generating Initial Codes: Descriptive codes were systematically assigned to meaningful segments of the data using NVivo-12. Two researchers (AM, JD) independently coded the transcripts, focusing on capturing both explicit (semantic) and underlying (latent) meanings. Discrepancies in coding were resolved collaboratively through discussion and consultation with the broader research team.
3. Identifying Themes: Codes were grouped into categories, and potential themes and subthemes were identified based on the study’s objectives and research questions. Patterns and connections between codes were carefully analysed to ensure they reflected the depth of the data.
4. Reviewing Themes: Preliminary themes were reviewed and refined to ensure they accurately represented the data and aligned with the study’s objectives. The broader research team (AM, AW, KU, JD) critically examined the themes to ensure coherence and data support. Redundant or overlapping themes were merged, and subthemes were added where appropriate.
5. Defining and Finalizing Themes: Each theme was clearly defined and described to ensure relevance to the study’s research questions. Themes were named to reflect their characteristics and aligned with the study’s theoretical framework where applicable.
6. Producing the Report: The final themes and insights were compiled into a comprehensive report contextualised within the existing literature. The supplementary file contains detailed data analysis (S1) and coding tree steps (S2).

### 2.6 Rigour

Trustworthiness is essential in qualitative research to validate the findings and enhance credibility [32]. In this study, every decision made during meetings was documented to ensure confirmability, dependability, and credibility across all phases of the research [33–35]. Participant quotes illustrate the findings, which multiple researchers analysed to ensure coder agreement [36,37]. NVivo12 was used for data analysis, enhancing rigour by validating or challenging the researchers’ initial interpretations of the data [38].

The first author (AM) conducted all interviews, with feedback from the other authors, who were all experienced qualitative researchers. Recognising the importance of reflexivity, AM kept a reflexive journal throughout the research process to reflect on personal assumptions, decisions, and interactions with participants, which could influence the data collection and analysis. This helped to identify potential biases and adjust the approach as needed.

Acknowledging the risk of interviewer bias, strategies were implemented to mitigate its influence. AM used open-ended, non-leading questions during interviews to encourage participants to express their experiences freely. Peer debriefing sessions with co-authors were held regularly to review transcripts and interpretations, ensuring that the findings were consistent with the data and not overly influenced by the interviewer’s perspectives.

Furthermore, coder triangulation was applied, where multiple researchers independently coded the data to ensure consistency and agreement on emerging themes.

### 2.7 Ethical considerations

Ethical approval was obtained from the relevant Human Research Ethics Committees at the University of Technology Sydney (Project ID (ETH22−7268) and the Ministry of Health in Saudi Arabia (IPR number H-02-K-076–07 22–775). Participants who expressed interest in being interviewed were provided with a participant information sheet outlining the study and requirements. Verbal recording consent was obtained from every participant before the interview, in alignment with the approved procedures by the IRB. The consent process involved reading the consent script to participants, confirming their understanding, and documenting their verbal agreement before proceeding with the interview. Each participant was fully informed about the research goals, scope, and rights, which included the option to withdraw from the study at any time without any negative consequences.

Participants were encouraged to ask questions during the interview and given enough time to ensure they fully understood the information before agreeing to participate. To maintain participant anonymity, pseudonyms were used during analysis and when reporting the results.

## 3. Result

### 3.1 Participant characteristics

A total of 13 participants were included in this study: (n = 10) Saudi and (n = 3) non-Saudi. There was one male and 12 female participants, which reflects the prevailing gender ratio in nursing in KSA. All participants held a bachelor’s degree, and two (2) had a master’s degree, which was the highest level of education in the cohort. The average age was 30 years, ranging from 25 to 40 years. The length of clinical experience ranged from 1.5 to 10 years, with an average of five years. The participants worked in inpatient wards (medical, surgical, intensive critical unit (ICU), cardiac critical unit (CCU), oncology, and emergency room (ER), paediatric) (Table 1).

**Table 1:** Participant characteristics.

Participant ID	Age	Gender	Nationality	Highest Degree	Years of Experience	Specialty
1	25	Female	Saudi	Bachelor	2.0	Medical
2	30	Female	Saudi	Bachelor	6.0	ER
3	22	Female	Saudi	Bachelor	1.5	Paediatric
4	28	Female	Saudi	Bachelor	4.0	Oncology
5	35	Female	Saudi	Master	10.0	ICU
6	27	Female	Saudi	Bachelor	5.0	CCU
7	24	Female	Saudi	Bachelor	2.5	ER
8	31	Female	Saudi	Master	7.0	Medical
9	29	Female	Saudi	Bachelor	5.0	Surgical
10	23	Female	Saudi	Bachelor	1.5	ICU
11	26	Female	Non-Saudi	Bachelor	3.0	ER
12	30	Female	Non-Saudi	Bachelor	6.0	ICU
13	32	Male	Non-Saudi	Bachelor	5.0	CCU

### 3.2 Result

**Fig 1:**
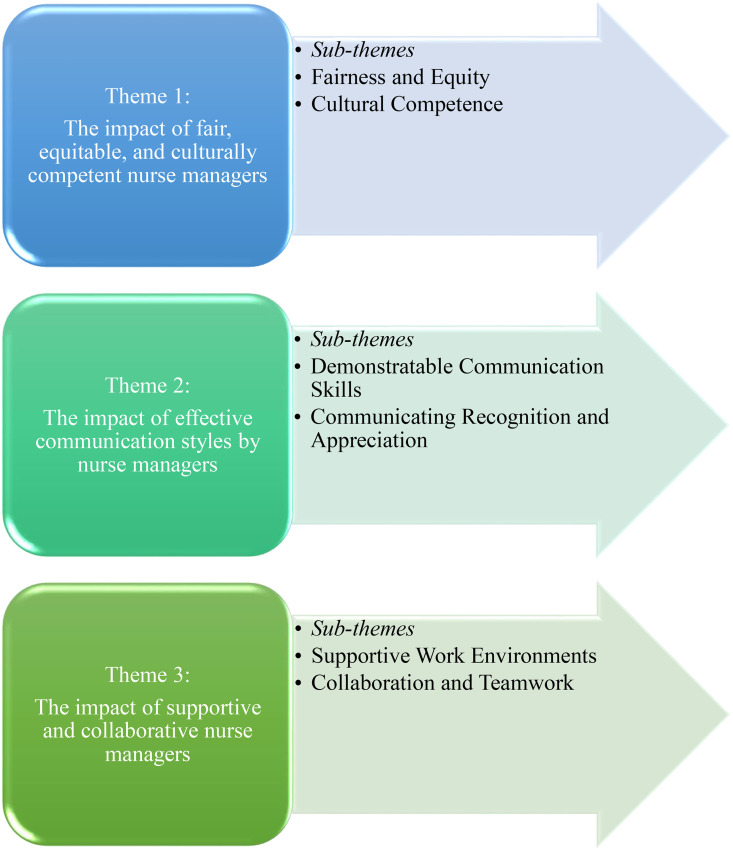
Thematic framework of the study.

Fig 1 outlines the thematic framework. The overarching themes are (1) the impact of fair, equitable, and culturally competent nurse managers, (2) the impact of effective communication styles by nurse managers, and (3) the impact of supportive and collaborative nurse managers. Six sub-themes, which support each overarching theme, are presented in this paper.

This figure illustrates the overarching themes and sub-themes describing registered nurses’ perceptions of nurse manager leadership and its impact on work engagement.

Fairness and equity, cultural competence, demonstrable communication skills, communicating recognition and appreciation, supportive work environments, and collaboration and teamwork. Each theme captures participants’ perspectives on the impact of observed and experienced leadership behaviours on their work experiences and views on work engagement.

### 3.3 Theme 1: The impact of fair, equitable, and culturally competent nurse managers

The participants highlighted the importance of fairness, equity, and cultural competence in leadership. They reported feeling more engaged and motivated when nurse managers treated all employees fairly, acknowledged their individual needs, and provided equal opportunities regardless of their background. Nurse managers who were culturally competent and respected the diverse cultural and ethnic backgrounds within their teams were commended for creating a positive and inclusive work environment.

#### 3.3.1 Fairness and equity.

Participants described specific leadership behaviours that demonstrated fairness and equity, making them feel more engaged at work. Conversely, participants discussed how unfair and unequal treatment from managers caused them to feel disengaged from their work.

Participants defined equitable treatment behaviours as those that acknowledge their unique contributions, needs, and circumstances in a manner free from bias or favouritism. These behaviours help create an environment where employees feel valued and treated fairly, positively impacting their engagement at work.

Abeer highlighted how her department manager’s approach to allocating tasks based on staff abilities and current workloads boosted her sense of engagement and belonging.: *“The head of our department allocated tasks and cases based on individual strengths and workloads, enhancing my engagement and sense of belonging*” (Abeer).

Huda also appreciated how their manager handled leave requests, taking everyone’s leave requests into account: *“Our head nurse considered our vacation needs, allocating leave fairly, which made us truly appreciate our work”* (Huda).

Participants felt that ‘fairness’ from managers motivated them to perform to the best of their abilities, knowing that their efforts and contributions would be appropriately recognised and appreciated: “*I enjoyed working with a wonderful head nurse in my current unit. The staff love her, and her approachable demeanour and equal treatment of everyone have made her a respected leader*,” adding “*She never uses her authority to make others feel inferior. When it comes to performance evaluations, you know she will be fair, which motivates us to work to our full potential.”* (Mona).

Participants said working with a nurse manager known for fair treatment fostered a culture of trust and openness. Abeer said, *“When this kind of leader gives any comment or suggestion, we accept it with open arms, knowing that this leader is fair and will not put them under pressure*” (Abeer).

Some participants identified the negative impacts of perceived unfair behaviour. For example, Nour described having lost leave days due to workload and not being compensated. She observed how this kind of treatment leads to decreased work engagement and increased absenteeism.

*We have a lot of compulsory leaves and days off... I can [theoretically]take 23 days off, but they … expired, and [the unit hasn’t] compensated us. Now, they don’t give us money for it; they give us extra time.... However, in [other] units, the head nurse arranges everything for the staff to take their days off. When we tried to talk to our head nurse, we were told there was a staff shortage... So, [there are] a lot of absences; [staff] don’t work with dedication”* (Nour).

Julia was not able to take breaks for breastfeeding, which affected her negatively. Despite continuing to fulfil her duties while breastfeeding her baby, the emotional toll this had produced a negatively cascading effect on her productivity at work:

*I was struggling to have my right to have a break for breastfeeding; this psychologically affected me at work. I used to work and give the patients everything they needed, but I was internally affected and disappointed... I was affected in terms of productivity. I don’t come to work actively as usual, smiling, and happy that I was coming to work. [While each] patient got all the medications they needed, I [did not do my best] work while I felt upset”* (Julia).

#### 3.3.2 Cultural competence.

Participants highlighted the importance of recognising and understanding religious and public holidays in their work practices, expressing disengagement when their managers failed to appreciate the cultural importance of these occasions. For example, Sarah highlighted a particular instance of cultural insensitivity: her manager did not recognise the significance of Eid, a major religious holiday, and expected her to remain at work during the initial days of the celebration, which are traditionally spent with family. This lack of understanding caused her to feel disengaged from her work:

*We spend most of our time together, having fun and laughing about non-work-related matters. Unfortunately, some managers don’t understand work-related issues. For instance, they may not realise that during Eid days, they expect you to stay with them for the first four or five days instead of returning to your family. This lack of understanding has led to many arguments……... I see it is just a job; even the idea of quitting crossed my mind. I no longer feel the same excitement and enthusiasm; I used to wake up hours before work and be excited and happy. Now, it’s merely a job; I hope to do it by the end of this period “*(Sarah).

Arwaa shared similar concerns about the lack of cultural sensitivity among non-Saudi managers towards national holidays:

*“… non-Saudi leaders don’t care about national vacations, the National Day, or the founding day. She doesn’t give these days off, no matter what happens … non-Saudi leaders don’t consider the cultural differences between Saudis and non-Saudis. As for the Saudi leaders, they are appreciative; they would give us our rights as Saudis … our country”* (Arwaa).

Arwaa also recounted personal experiences of being unable to meet with family due to work demands:

*She used to ask me to work every weekend. I told her that I couldn’t come for a whole month. I can’t meet with my family. I can’t go anywhere else. When I tried to talk to her about this issue, she didn’t understand it. She says we all have families. …There are many absences and sick leaves. I mean, it is normal for someone to put pressure on the staff, but they won’t tolerate it. They won’t be able to bear this anymore”* (Arwaa).

Participants discussed how managers’ lack of cultural competence, especially non-Saudi leaders, resulted in biased treatment that negatively impacted team morale. Nour shared her experience with a manager who showed favouritism toward individuals from the same nationality, undermining her sense of inclusion and engagement:

*Sometimes, the non-Saudi leader tends to be biased toward her group; she treats them much better than other nationalities. She tends to discriminate between nationalities, like racism. Because they are the same nationality, they get better treatment. For example, if they have requests, she agrees and makes things easier for them, like schedules. But when I, as a Saudi woman, asked for something, she told me, “No, I can’t.” These are the main problems”* (Nour).

Participants explained that this cultural insensitivity not only created frustration but also increased absences and sick leave, contributing to burnout among the staff. Nour further highlighted how these practices eroded engagement:

*There are many absences and sick leaves. When managers treat people unfairly and don’t understand the importance of fairness across cultures, staff can’t tolerate it. They stop caring about the work. I had completed my first year at work and already felt burned out”* (Nour).

Another participant highlighted the need for nurse managers to maintain objectivity and not show favouritism, citing how this bias negatively affected the team’s desire to work: *“They should treat all employees equally. They shouldn’t specially treat friends or be biased toward anyone… [otherwise, others] would no longer want to go to work”* (Khalid).

### 3.4 Theme 2: The impact of effective communication styles by nurse managers

Participants emphasised the importance of effective communication and how nurse managers can engage their teams through active listening, verbal affirmations, and two-way communication in which nurses are encouraged to participate in decision-making processes. Participants also highlighted the positive impact of a nurse manager’s ability to provide recognition and appreciation to staff.

#### 3.4.1 Demonstratable communication skills.

Participants emphasised the critical role of effective communication from leaders in fostering a sense of dedication and involvement among nursing staff. They particularly valued being heard, illustrating how active listening by nurse managers can significantly enhance workplace dynamics. Abeer highlighted the responsiveness of her head nurse to concerns, stating, *“When a problem arises, the head nurse attentively listens to our issues and seeks solutions. This support enables us to excel in our tasks, deepening our commitment to work”* (Abeer).

Huda said she felt more valued and less stressed when her manager discussed relevant issues with staff, holding brief meetings to address problems and provide detailed guidance:

*She often discusses every point with her staff. She would hold brief meetings with them to discuss issues and guide them in detail on tasks such as sending emails and receiving cases. There is communication. Despite the high workload in this unit, handling 8-9 cases per shift, the staff feel less stressed than those in other units with lower capacities”* (Huda).

Study participants appreciated nurse managers who considered individuals’ personal needs, and this fostered a strong connection between staff members and their work. May articulated this, stating:

*“Leaders who listen to our opinions consider what’s best for the team. Our head nurse always consults us, even about scheduling, to accommodate our events and preferences. This approach respects our rights and needs and makes us feel valued and considered”* (May).

Participants highlighted the importance of nurse managers who listened and addressed concerns, especially regarding work related stress. They emphasised that these kinds of issues required serious consideration and genuine support from leadership. There was a strong consensus that employee satisfaction correlates to high-quality patient care, emphasising the value of feeling supported and appreciated in their roles. Jolia said:

*“[the manager] should listen to the employees; for example, if the employees have some problems, [the manager] should try to solve them, not ignore them; even if the employee is going through … job burnout, [the manager] should take it seriously. The leader should prioritise making employees feel happy and satisfied to help patients properly”* (Jolia).

#### 3.4.2 Communicating recognition and appreciation.

Managers who expressed appreciation of staff fostered greater work engagement and commitment in their team:

*When a leader appreciates his employees, gives them their rights, and respects them, it makes a great difference…. I come to work with love, feeling happy and enjoying working with her”* (Sarah).

Participants expressed how recognising the diverse needs of nursing staff is vital as it influences their energy and enthusiasm for work. Jody’s statement highlighted this:

*“It’s different from one staff member to another. While some are motivated by self-development, others appreciate small gestures like coffee or sweets. I seek a balance in my workload and value cooperation when I need a vacation; [then] I can do more work”* (Jody).

Mona and Jolia highlighted the significant role of appreciation in managing work pressures and encouraging extra effort. Mona stated, *“When leaders appreciate your work, you can work properly and bear all the pressure at work because there is appreciation*.”

Jolia echoed this: *“If a good leader needed me for extra work, I would do whatever they asked. The appreciation [would] make me willing to take overtime and add new tasks [especially where they create] a comfortable, safe, and good work environment*”(Jolia).

Nour and Khalid both felt that genuine appreciation outweighed financial incentives, underscoring the deep impact of recognition on staff engagement: “*When you know someone appreciates your work, you will find people working with dedication. They work from their heart, not just for a salary”* (Nour). Khalid also supported this: *“If manager leaders are appreciative, we will be interested in getting involved in and engaged in our work”* (Khalid).

Another participant highlighted how expressions of recognition, such as positive appraisals, salary increments, and incentives, directly contributed to making the physical and emotional stresses of the workplace more bearable:

*“We will be more engaged if the manager is appreciated. Good appreciation appraisals, increments, and incentives make physical and emotional stress manageable because we feel recognised and rewarded.”* (Abeer).

In contrast, a lack of appreciation can have detrimental effects on staff engagement: *“They realise no one appreciates their efforts, so why should [they] work with all [their] heart? They [leadership] won’t appreciate it”* (Mona). Nour shared her experience with a head nurse: *“In the Paediatrics unit, a head nurse known for harsh orders and a lack of listening skills faced widespread resistance, for example: “Most nurses tended to ignore her orders, resulting in absences and a severe staff shortage”* (Nour).

### 3.5 Theme 3: The impact of supportive and collaborative nurse managers

Supportive and collaborative leadership was perceived as having a positive impact on staff engagement. Participants shared experiences of how nurse managers who were supportive and collaborative facilitated teamwork and provided emotional and professional support, enhancing team dynamics, morale, and overall work engagement.

#### 3.5.1 Leadership creating the supportive work environment.

One participant noted that nurses feel supported if, when they are feeling stressed or going through a tough time, and their manager takes an empathetic approach and provides support. This approach fosters a familial atmosphere and encourages staff to stand together during difficult periods:

*If a nurse comes to work stressed or faces a hard time, the head nurse asks her what’s wrong and then supports her. She asks other nurses to stand with her, assigning her small tasks with stable cases, not critical ones. This way, we all feel satisfied, more engaged, and dedicated to our work and each other, knowing we could be in this situation one day and the head nurse would stand with us. We are like a family in one unit”* (Julia).

Nurse managers who support their teams, especially during periods of conflict, such as disagreements between staff members or other health workers, gain respect and loyalty from their staff. Nurse managers who modelled supportive behaviour encouraged team members to support each other:

*Our leader consistently supports us, even in conflicts with other professionals. Her unwavering support has made us deeply loyal; no one wants to leave her unit. We all recognise her value and reciprocate her support, ready to assist whenever she needs us”* (Arwaa).

Similarly, a supportive leader positively impacted the team’s ability to manage their workload and challenges, especially in challenging circumstances. Mona described this as follows: *“The most motivating factor for me is knowing that my leader is always there to support and guide me, especially in critical situations. [Their] expertise and availability significantly affect how we handle our workload and challenges”*(Mona).

Participants felt that having nurse managers who promoted cooperation and mutual support created a better work environment, resulting in lower absenteeism and a stronger sense of community.

*The labour ward at our hospital operates like a family, emphasising teamwork and support. Our manager encourages us to assist busy colleagues and be attentive to each other’s needs. This collaborative environment has resulted in low absenteeism; typically, only two people might be absent per shift, and they always communicate their reasons. Our leader’s human and attentive approach, always ready to listen, fosters this strong sense of community”* (Samar).

Samar further noted as follows: “*Even when we are sometimes busy, she comes in the morning and asks us if we had breakfast. She tells us to leave everything in our hands and eat before starting work”* (Samar).

#### 3.5.2 Collaboration and teamwork and their impact on nurses.

Participants said that the way nurse managers treat their teams significantly impacts engagement and motivation. Leaders who ensured their staff’ rights were protected, for example, positively influenced staff attitudes towards work and were respected and valued:

*A strong connection exists between a leader’s treatment of their employees and the level of staff work engagement. When a leader shows appreciation, grants employees their rights, and treats them respectfully, it can make a significant difference. For example, when I work in a department with a collaborative leader, I come to work with love; I feel happy and motivated to work with them”* (Nour).

Conversely, a work environment where employees feel uncomfortable, unsafe or lack agency results in a deterioration of genuine commitment to their roles:

*If the leader has a good relationship with the employee, the employee will have more interest and benefit from team spirit. When the employee has team spirit, they will love their work and dedicate their time to it. This dedication must be physical and mental. However, if the employee is uncomfortable and does not feel safe and was only taught to do the work and go home, whatever they do at work becomes unimportant, so I do not think this person becomes engaged as required”* (Huda).

Participants spoke of supportive leaders they have encountered in the past. For instance, a previous head nurse with a kind and collaborative nature fostered respect and motivation, resulting in a positive work environment. On the other hand, head nurses who lacked these qualities had a negative impact on the team. This led to increased absenteeism and diminished work quality, as their inability to inspire and support their team directly affected staff enjoyment of and commitment to their work:

*When I first came here, I noticed that the staff admired and respected one of the previous head nurses, and until now, they love and praise him, and they wish he would be with them because he was so kind and collaborative. He was such a good leader. When I observe a head nurse in another unit, I notice how well he collaborates with the staff. [He] motivates them and supports every step they take; it makes me wish for a head nurse like him in my unit. In contrast, I have seen some head nurses in other departments who do not motivate their staff or provide support, leading to increased absences and decreased work quality. The leader’s influence on their team is crucial, as it can make or break their love and dedication for the unit and their job”* (Samar).

Khalid believed that the absence of a collaborative spirit, especially between management and staff, breeds dissatisfaction through lack of fulfilment. As a result, the staff feel trapped in their roles and work solely for personal goals such as financial needs or career advancement rather than fostering a shared commitment to the workplace:

*As a manager and staff member, we have never had this type of teamwork. Most of our staff are not satisfied. We keep working because every one of us has a goal; some of us work for the sake of money, people work because they want to reach something bigger, while others work to earn a living, so they get stuck in this place”* (Khalid).

### 3.6 Staff nurses’ understanding of staff engagement

Participants were asked about their understanding of work engagement. Most were initially unfamiliar with the term “work engagement” or struggled to define it. The researcher then introduced the key components of work engagement – vigour, dedication, and absorption – and provided brief definitions to help the nurses relate these concepts to their experiences.

During these discussions, spirituality emerged unprompted as a central motivational factor, despite not being explicitly mentioned by the researcher. Participants shared that their spiritual beliefs deeply influenced their dedication to their work. It became evident that for many participants, spirituality was deeply intertwined with their motivation and commitment to their roles.

For instance, Abeer highlighted the impact of spirituality on her work ethic, stating, “*The fear of God motivates me the most. It compels me to excel in my job, driving my engagement at work.*” Similarly, Nour emphasised her spiritual dedication, “*I give my all, working full-time with utmost dedication. Even knowing my efforts might not be acknowledged, I work for the sake of God, pouring my heart and soul into my duties*”.

These responses indicate that spirituality played a key role in shaping their understanding of engagement, providing them with a sense of purpose and resilience in their daily work. However, participants did not attribute this spiritual motivation to their managers’ actions or behaviours. Instead, they viewed spirituality as an intrinsic factor guiding their commitment to their professional responsibilities.

## 4. Discussion

This study explored participants’ perspectives on how their managers’ leadership practices impact work engagement. Participants emphasised the significance of leadership qualities such as fairness, equity, cultural competence, effective communication, and a supportive and collaborative approach in fostering employee engagement and creating a positive workplace environment.

Participants highlighted the importance of fairness and equity in promoting work engagement. Nurse managers can foster this by applying transparent decision-making processes, fair workload distribution, and equal opportunity for career growth. This aligns with existing research that recognises fairness as a critical motivator for enhancing a sense of belonging in the workplace [39]. Studies conducted in Western countries and other healthcare settings have similarly identified fairness as essential for staff engagement and job satisfaction [40]. Additionally, a study conducted in Iran emphasised that fairness in leadership is a fundamental driver of employee well-being, workplace success, and job satisfaction [41]. Their findings suggest that employees in fair work environments experience higher engagement, resilience, and professional commitment, reinforcing the idea that fairness is not only an ethical obligation but also a strategic necessity in leadership.

The importance of perceived fairness extends beyond individual success, influencing how employees manage job demands and impacting patient outcomes [40]. Conversely, a lack of fairness can increase staff anxiety [41], reduce enthusiasm for work [42], and cause emotional exhaustion [40]. The study from Iran further supports this perspective, indicating that workplace injustices contribute to disengagement, decreased morale, and lower commitment to organisational goals [41].

To promote fairness, it is crucial to ensure equitable treatment, eliminate biases, and encourage impartial conduct in all aspects of the work environment, including interactions with colleagues, distribution of benefits, and professional relationships [43]. Furthermore, fairness is important to mitigate burnout among nurses [44,45]. Inconsistencies in resource allocation, rule application, and perceived workplace status negatively affect participants’ job satisfaction and mental health [45].

This study highlights the importance of nurse managers creating a culture of understanding and respect for the diverse cultural backgrounds of their staff. Nurse managers need to consider the individual needs of different genders, ages, ethnicities, and cultural backgrounds when leading a diverse workforce [46,47]. Cultural competence in nursing leadership is crucial in diverse settings such as Saudi Arabia, where nurses come from many different cultural backgrounds [47–50].

The participants in this study recognised the significance of creating an environment where cultural differences are celebrated, including understanding religious and public holidays, and ensuring an inclusive environment for staff, irrespective of their background, to ensure a sense of inclusion. This is consistent with existing literature that recognises the significance of cultural nuances in fostering a positive and supportive atmosphere where every individual feels valued [49]. Such awareness allows nurse managers to effectively meet their staff and patients’ diverse needs and increase satisfaction, trust, and overall organisational performance [46,51].

This study extends existing knowledge by underscoring the role of religious inclusivity as a critical component of cultural competence. Participants reported recognising religious practices and ensuring accommodations significantly enhance staff morale and motivation. While previous research has explored ethnic and linguistic diversity in leadership [47,48], fewer studies have examined how religious considerations influence leadership effectiveness in healthcare settings. Given that Saudi Arabia integrates cultural and religious values into both professional and personal life, these findings highlight the importance of holistic cultural competence that encompasses both social and religious dimensions of diversity.

Nurse managers who embrace and integrate diverse cultural perspectives can foster a more inclusive, supportive, and effective care environment [46,51]. This, in turn, enhances patient care outcomes and boosts staff morale, reinforcing trust and engagement across the organisation [47]. Conversely, nurse managers lacking cultural sensitivity diminish workplace engagement and risk creating an environment of bias and discrimination, leading to a lack of unity and increased workplace dissatisfaction [47]. These findings reinforce the need for **structured cultural competence training** in leadership development programs. By equipping nurse managers with the skills to lead diverse teams effectively, healthcare organisations can cultivate a more cohesive, engaged, and high-performing workforce.

A unique and unexpected finding of this study is the role of personal spirituality in nurse engagement. Although the participants were not directly asked about their spirituality, they revealed that their belief in God is essential to their work ethic and motivation. This highlights an underexplored dimension of work engagement that has not been extensively addressed in nursing leadership literature. Most existing research on spirituality in healthcare focuses on how nurses provide spiritual care to patients [25,52,53], while this study shifts the focus to how nurses’ personal spirituality influences their professional engagement.

Given that spirituality plays a central role in Saudi society, its impact on work engagement deserves further exploration. More research is needed to understand how spirituality influences work engagement in the Saudi cultural context, in which professional and spiritual lives are deeply connected.

## 5. Implications

### 5.1 Implications for practice

The findings highlight the importance of leadership traits such as cultural sensitivity, inclusivity, and fairness in fostering supportive and engaging work environments. These traits align with Saudi Vision 2030 by promoting the retention of Saudi nurses, reducing reliance on expatriate staff, and contributing to a resilient healthcare workforce. Furthermore, these leadership practices directly support Vision 2030 by enhancing patient care quality, empowering Saudi nurses, advancing workforce diversity, and positioning Saudi Arabia as a leader in healthcare excellence.

Currently, limited research has explored the effectiveness of leadership training tailored to Saudi Arabia’s culturally diverse nursing workforce [48]. Therefore, Leadership development programs for nurse managers should prioritise cultural competence, effective communication, and fairness in decision-making to enhance staff engagement and retention. Targeted interventions, such as implicit bias training, communication workshops, and leadership simulations, can equip nurse managers with skills to support a diverse workforce while maintaining equitable practices. These strategies enhance retention, reduce burnout, and improve patient care quality.

Healthcare organisations should integrate team-building initiatives to foster mutual respect and cultural awareness [55]. Cultural competence training should also be prioritised to reduce bias, enhance cross-cultural communication, and improve workforce cohesion [56]. By strengthening leadership capacity among Saudi nurse managers, these strategies help create a self-sustaining nursing workforce, reducing reliance on expatriate staff while fostering a stable and engaged team aligned with Saudi Vision 2030’s workforce localisation objectives.

### 5.2 Implications for future research

This study provides a foundation for future research on cultural competence training and its adaptability in diverse healthcare systems. The findings encourage nurse managers to refine leadership strategies to enhance engagement and retention, supporting Saudi Vision 2030’s workforce goals. Future studies should explore the long-term impact of cultural competence training on nurse engagement and leadership effectiveness. Comparative research across healthcare systems can assess how leadership strategies adapt to different cultural and organisational settings. Further investigation into spirituality’s role in professional motivation may provide insights into its effect on nurse retention and leadership perceptions. Longitudinal studies are recommended to assess the sustained impact of leadership interventions on nurse retention, workforce stability, and healthcare service quality.

## 6. Limitation

While the qualitative approach enabled in-depth insights from participants, the research was confined to four hospitals in the western region of Saudi Arabia, potentially limiting the generalisability of the findings. Additionally, participants may have been hesitant to critique leadership due to cultural norms, which could introduce some biases in their responses.

While most participants were women, this reflects the female dominance in the nursing healthcare sector in Saudi Arabia. Another limitation is that the study focused solely on the perspectives of nurses. Including the perspectives of nurse managers could provide a more comprehensive understanding of the interplay between leadership practices and nurse engagement. Further research in varied settings is recommended to enhance understanding of this phenomenon.

## 7. Conclusion

This study found that nurse managers’ leadership practice significantly affects nurses’ work engagement in Saudi Arabia. Leadership qualities such as fairness, cultural competence, effective communication, and supportive leadership are pivotal to enhancing work environments. By fostering these qualities, healthcare facilities can enhance nurse engagement, staff retention and patient care outcomes. Future research could benefit from longitudinal studies that track the implementation of specific leadership interventions over time and assess their sustained impact on nurse engagement and patient outcomes. Additionally, comparative studies examining leadership styles across different cultural and healthcare systems would provide deeper insights into how leadership approaches can be adapted to diverse contexts. Also, Further research is recommended to explore the impact of personal spirituality and cultural nuances on work engagement to tailor leadership strategies effectively in culturally diverse environments.

## Supporting information

S1 File Detailed data analysis. This file contains comprehensive data analysis steps.
(DOCX)

S2 File Coding tree steps. This file outlines the steps used in the coding process, clearly detailing how the themes and categories were developed from the data.
(DOCX)
