# Supplementary material for: Exploring registered nurses’ experiences and perceptions of nurse manager leadership and its impact on work engagement: A qualitative study set in Saudi Arabia
Source: PLoS One. 2026 Feb 13;21(2):e0340471. doi: 10.1371/journal.pone.0340471 (PMC12904394; doi:10.1371/journal.pone.0340471)
Supplement: S1 File — This file contains comprehensive data analysis steps. (DOCX) [file pone.0340471.s001.docx]

**Data analysis steps:**

| **Action** | **What Happened** | **Relevance** |
| --- | --- | --- |
| Initial reflections captured in a diary (AM) | Post-interview, AM took notes and researched reflections. This served to reflect on the challenges in the interview process and capture any initial stories or ideas that AM was forming. | Data familiarisation Rigour Positioning |
| First cross-checking of transcripts against audio (AM) | Transcripts were completed for the English interview and reviewed while listening to the interview audio. AM corrected the audio after re-listening to it. Initial thoughts were captured during the re-listening. | Data familiarisation Rigour Positioning |
| Translation | After review, a bilingual translator translated all Arabic transcriptions into English. | Data familiarisation Rigour Positioning |
| Independent review of all transcripts to capture compelling stories or sections of interest (AM and JD) | AM and JD shared transcripts/translations through NVivo. | Data familiarisation Rigour Use of scripting convention. |
| Transcripts are read independently, and first thoughts or initial codes are captured (AM and JD) | AM and JD independently read the transcripts, capturing initial thoughts and codes. They discussed similarities and differences in their coding during multiple meetings, consolidating initial codes and considering how different codes could form overarching themes.  Were captured. Initial codes from both AM and JD were captured on NVivo. | Data familiarisation Generation of codes. |
| Grouping of themes meeting (AM and JD) | AM and JD met five times to discuss the initial codes and consider groupings. AM logged responses onto NVivo. | Combining codes into themes. Theme development meeting (JD and AM) Initial themes were grouped and reviewed against the study aims. |
| Theme development meeting (AM, JD, AW, KU) | Initial themes were grouped and reviewed against the study aims.  AM met with JD, AW and KU to discuss their reflections and review initial themes against study aims. | Reviewing themes Determining the significance of themes Establishment of themes |
| Establishment of Themes (AM, AW, and KU | AM, AW, JD, and KU collaboratively reviewed the developed themes to ensure they aligned with the research questions and were supported by the data. Adjustments were made to refine the specificity and inclusiveness of the themes. | Ensuring themes are robust, applicable, and reflect the data comprehensively. |
| Reporting of results | The established themes and insights were compiled into a comprehensive report, and discuss the findings with existing literature. This report was prepared for peer review and included detailed examples and direct quotes from the data to illustrate the themes. | Communication of findings Impact on the field |
