# Supplementary material for: Exploring registered nurses’ experiences and perceptions of nurse manager leadership and its impact on work engagement: A qualitative study set in Saudi Arabia
Source: PLoS One. 2026 Feb 13;21(2):e0340471. doi: 10.1371/journal.pone.0340471 (PMC12904394; doi:10.1371/journal.pone.0340471)
Supplement: S2 File — This file outlines the steps used in the coding process, clearly detailing how the themes and categories were developed from the data. (DOCX) [file pone.0340471.s002.docx]

- He should treat all employees equally.
- The tasks should be divided equally.
- Competitions are supposed to be fair.
- I was struggling to have my right to have a break from breastfeeding.
- Her approachable demeanour and equal treatment of everyone make her a respected leader.
- When employees feel that they are all equal, they will be creative and reassured.
- The employee must feel he is being treated fairly according to different nationalities and qualifications.
- Non-Saudi leaders don’t care about national vacations.
- Saudi leaders are more understandable.
- Non-Saudi had team cohesion and support.
- Unfortunately, they don’t understand work-related issues; for example, they may not realize that during Eid days.
- The non-Saudi leader tends to be biased toward her group.
- When a decision is made, the leaders are afraid. They don’t have a strong personality to say no.

**Fairness and Equity**

**Cultural Competence**

- A good leader should have good communication skills and give complete support to his staff.
- Before deciding such as overtime or time back, he would consult with the staff.
- Communication really affected.
- She comes in the morning and asks us if we had breakfast.
- Making us feel like she is one of us.
- A good leader should be willing to take, give, and listen to others.
- Essential to gather the opinions of those working with me and then see what's best.
- I prefer the kind who discusses everything with the staff.
- The method of communication makes a difference.

**Communicating Recognition and Appreciation**

**Demonstrable Communication Skills**

- When you know someone appreciates your work, you will find people working with dedication.
- Verbal praise by mere words.
- Recognition of employee rights.
- With kind words, greeting and smiling to the staff, and speaking and asking about them, this gives the biggest positive energy to the staff at the beginning of the day.
- If manager leaders appreciate, we will be interested in getting involved.
- The leader should give the employee his rights and grant him appreciation.
- Appreciation like bringing a cup of coffee or any type of sweets.
- Diversify different types of incentives.
- Provide rewards and acknowledge the efforts of a team.

.

**Collaboration and Teamwork**

- She encourages collaboration and teamwork.
- Treating us like we were one family.
- I work in a department with a collaborative leader, I come to work with love; I feel happy and motivated to work with them.
- we have never had this type of teamwork. Most of our staff are not satisfied.
- When the employee has team spirit, he will love his work and dedicate his time to it.
- Staff need a supportive work environment.
- Stand with staff, not against them.
- As a leader, I would try to observe nurses' weaknesses to fix them. Then I would observe the strengths points of nurses.
- Support during stressful seasons.
- Our unit consistently received top grades and positive evaluations.
- Maintain a positive relationship with staff.

**Supportive Work Environments**
